# Association between growth differentiation factor 5 rs143383 genetic polymorphism and the risk of knee osteoarthritis among Caucasian but not Asian: a meta-analysis

**DOI:** 10.1186/s13075-020-02306-9

**Published:** 2020-09-14

**Authors:** Lei Peng, Song Jin, Jiping Lu, Chao Ouyang, Jiang Guo, Zhongyu Xie, Huiyong Shen, Peng Wang

**Affiliations:** 1 Department of Orthopedics, The Eighth Affiliated Hospital, Sun Yat-sen University, 3025# Shen Nan Road, Shenzhen, 518033 People’s Republic of China; 2 Department of Orthopedics, The Second Affiliated Hospital, Sun Yat-sen University, 107# Yan Jiang Road West, Guangzhou, 510120 People’s Republic of China; 3 Department of Orthopedics, The Second Affiliated Hospital of Hunan Normal University, The 921 Central Hospital of the People’s Liberation Army, Hongshan bridge, Changsha, People’s Republic of China

**Keywords:** Knee osteoarthritis, Polymorphism, Growth differentiation factor 5, rs143383, Caucasian

## Abstract

**Background:**

A few months ago, the *Bioscience Reports* journal showed that growth differentiation factor 5 (GDF5) rs143383 genetic polymorphism increases the susceptibility of knee osteoarthritis (KOA), but previous studies’ results have debates about available data. Considering the availability of more recent data, we focus on clarifying the relationship of KOA and GDF5 rs143383 genetic polymorphism by a meta-analysis of case-control trial data.

**Methods:**

The eligible studies from the time of database established to Oct. 2019 were collected from PubMed, Springer, Cochrane library, Web of Science, China National Knowledge Infrastructure (CNKI), and Wan Fang library. Odds ratios (OR) and 95% confidence intervals (CI) were used to estimate the association between these polymorphisms and KOA risk. The meta-analysis was completed by STATA 18.0 software.

**Results:**

A total of 196 studies were collected, 16 of them included in final meta-analysis (7997 cases and 12,684 controls). There was significant association between GDF5 rs143383 polymorphism and KOA in all genetic models (for Allele model (C versus T): OR = 0.84 (95% CI = 0.76–0.91); dominate model (CC+CT versus TT): OR = 0.80 (95% CI = 0.72–0.90); recessive model (CC versus CT+TT): OR = 0.79 (95% CI = 0.68–0.92); heterozygote model (CT versus CC+TT): OR = 0.89 (95% CI = 0.80–0.97); homozygous model (CC versus TT): OR = 0.71 (95% CI = 0.60–0.85)). In the subgroup analysis, we obtained the results that there is no significance among Asians.

**Conclusion:**

GDF5 rs143383 genetic polymorphism increases the risk of KOA among Caucasians; CC genotype and C allele are protective factors for the susceptibility of KOA among Caucasians.

## Introduction

Osteoarthritis (OA) is a chronic degenerative disease and is a very important factor for disability in worldwide [1–3]. OA involves the knee, hip, wrist, etc. Knee osteoarthritis (KOA) is a common degenerative joint disease among osteoarthritis diseases [4]. Knee osteoarthritis is a multi-factorial disease, and its pathogenesis is currently unclear. Current studies have shown that smoking, diet, exercise, and genes are all associated with osteoarthritis [5]. The diagnosis of knee osteoarthritis is currently mainly diagnosed by imaging. There is no good way to predict the incidence of knee osteoarthritis. Recently, more and more attention has been paid to the study of knee osteoarthritis genes. It may not only explain the problem in genetics but also provide strong treatment directions for clinical workers.

Growth differentiation factor 5 (GDF5) rs143383 genetic polymorphisms is the association of the susceptibility of knee osteoarthritis reported in *Bioscience Reports* [5]. GDF5, also known as cartilage derived morphogenetic protein 1 (CDMP1), is an extracellular signaling molecule that participates in the development maintenance and repair of bone, cartilage, and other tissues of the synovial joint, with penetrant and rare deleterious mutations of the GDF5 gene resulting in dominant skeletal defects [6]. GDF5 is overexpressed in articular cartilage relative to osteophytic cartilage of OA patients, suggesting that it may play an important role in maintaining the stable articular chondrocyte phenotype [7].

Although the association of the risk of knee osteoarthritis and GDF5 rs143383 genetic polymorphisms has been described in several meta-analyses [8–12], several recent trials also reported the risk of knee osteoarthritis and GDF5 rs143383 genetic polymorphisms [4–6]. On the other hand, prior meta-analyses have some limitations, such as low quality and lacking enough studies. Therefore, we needed to update the data of the relevant studies and aim to clarify the relationship of GDF5 rs143383 genetic polymorphisms and the risk of knee osteoarthritis. Our article showed a latest and comprehensive relationship of GDF5 rs143383 genetic polymorphisms by using the latest and comprehensive case-control studies (Registered number: CRD42020168180, http://www.crd.york.ac.uk/prospero/).

## Methods and study designs

The eligible studies from the time of database established to Oct. 2019 were collected from PubMed, Springer, Cochrane library, Web of Science, China National Knowledge Infrastructure (CNKI), and Wan Fang. Two independent authors extracted the data and assessed case-control trial quality.

In the meta-analysis, we made use of the Newcastle–Ottawa Scale in Epidemiology (NOS) group [13]. The PubMed, Springer, Cochrane library, Web of Science, China National Knowledge Infrastructure (CNKI), and Wan Fang library were searched (updated to October 20, 2019) with terms “differentiation factor 5,” “GDF5,” “rs143383,” “polymorphism,” “osteoarthritis,” and “OA,” as both medical subject heading (MeSH) terms and text words to find all papers that had studied the association of GDF5 with OA. A manual search was applied to finding unknown references to additional studies. English and Chinese language restrictions were applied. Studies were selected if they satisfy the following criteria: (1) case-control study; (2) sufficient published data for calculating the odds ratio and 95% confidence interval; (3) the association of GDF5 polymorphism with OA; (4) matched Hardy–Weinberg equilibrium (HWE) in control cases; and (5) having five models’ data of allelic model, homozygote model, heterozygote model, recessive model, and dominant model.

### Data extraction and assess of quality

Two researchers (Lei Peng and Jiping Lu) conducted eligible studies based on the above inclusion criteria and collected information on each eligible study according to the inclusion criteria. The following items were extracted: first author, year of publication, country, population, genotype distribution, Hardy–Weinberg equilibrium (HWE), case, and control size. To avoid the wrong data, the researchers will examine the collected data and make a conclusion through discussion. The quality of studies was evaluated by two independent investigators (Peng and Lu) based on the Newcastle–Ottawa Scale (NOS) for case-control studies [14]. The study was considered high quality with the scores were ≥ 7. In the case of disputes, we settle disputes through discussion. A third investigator (Peng Wang) decided this on the basis of discussions.

### Statistical methods

Pearson’s *χ*^2^ test estimates deviation from HWE in the control group according to genotype distributions. Crude OR with their 95% CI was estimated and used to assess the strength of association between GDF5 rs143383 polymorphism and KOA. The pooled OR was calculated respectively for allelic effect of C versus T, homozygote comparison of CC versus TT, heterozygote comparison of CT versus CC+TT, recessive model (CC versus TT+CT) and dominant model (CC+CT versus TT). The significance of the pooled OR was determined by the *Z*-test (*P* ≤ 0.05). *Q* statistics (*P* < 0.10) indicated the evidence of heterogeneity was used to assess heterogeneity between studies. When significant heterogeneity was achieved (*P* < 0.10), the effect size of the study was combined with the random effect model; otherwise, the fixed effect model was used. Subgroup analysis was performed according to population, and sensitivity analysis was performed to determine the impact of individual studies on the aggregated results and to test the reliability of the results. The potential publishing bias was estimated by Begg’s funnel plot and Egger regression test. All cases were analyzed by STATA 18.0 software (Stata Corporation, College Station, TX, USA). The *P* values were bilateral. This study followed the PRISMA standard.

## Results

### Studies extraction and characteristics of studies

**Fig. 1:**
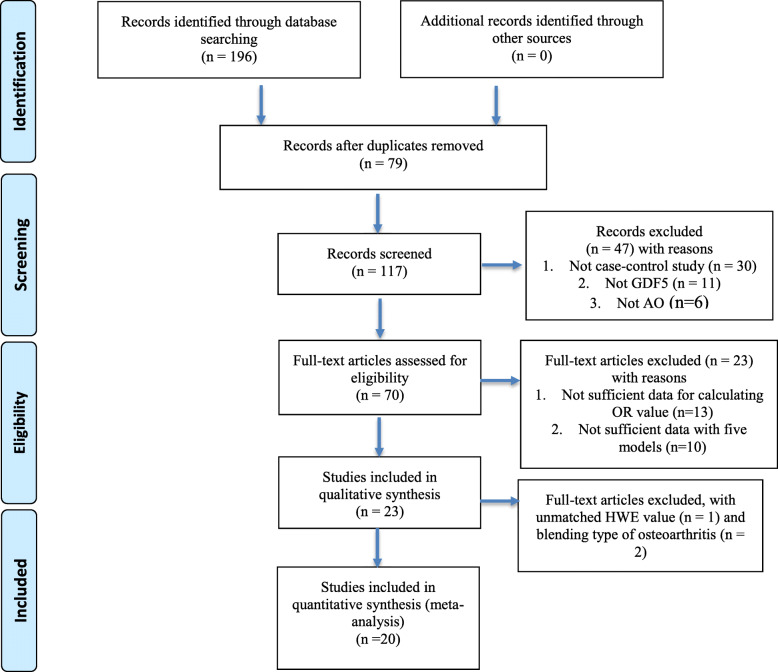
Studies identification diagram (from [13])

Sixteen articles [5, 11, 15–28], including separate 20 studies (7997 cases and 12,684 controls), finally have been collected in the meta-analysis from 196 studies, which process is showed in Fig. 1. All studies’ details, including first author name, year of publication, ethnicity, country, sex, gender ration, evaluation of quality (NOS), HWE, study design, genotyping method, and mean age, are shown in Table 1.

**Table 1:** Relevant studies concerning relationships between GDF5 rs143383 polymorphisms and knee osteoarthritis

Author	Year	Country	Ethnicity	Type of disease	Study design	Sample size of case/control	Genotyping methods	HWE among controls	NOS
Elazeem et al. [16]	2017	Egypt	Caucasian	KOA	Case-control	50/50	Taqman	0.58	8
Ozcan et al. [17]	2016	Turkey	Caucasian	KOA	Case-control	94/279	PCR-RFLP	0.06	8
Mishra et al. [15]	2013	India	Asian	KOA	Case-control	300/300	PCR-RFLP	0.97	8
Tawonsawatruk et al. [19]	2011	Thailand	Asian	KOA	Case-control	90/103	PCR-RFLP	0.63	8
Cao et al. [20]	2010	Korea	Caucasian	KOA	Case-control	276/298	PCR-RFLP	0.34	8
Valdes et al.—Nottingham [21]	2009	UK	Caucasian	KOA	Case-control	735/654	Allele-specific PCR	0.99	8
Valdes et al.—Chingford [25]	2009	UK	Caucasian	KOA	Case-control	259/509	Allele-specific PCR	0.90	8
Tsezou et al. [22]	2007	Greece	Caucasian	KOA	Case-control	251/268	Direct sequence	0.66	8
Miyamoto et al.—Japan [18]	2007	Japan	Asian	KOA	Case-control	718/861	Taqman	0.96	8
Miyamoto et al.—China [18]	2007	China	Asian	KOA	Case-control	313/485	Taqman	0.28	8
Southam et al.—UK [23]	2007	UK	Caucasian	KOA	Case-control	509/822	PCR-RFLP	0.26	8
Southam et al.—Spain [23]	2007	Spain	Caucasian	KOA	Case-control	274/1196	Taqman	0.54	8
Shin et al. [24]	2012	Korea	Asian	KOA	Case-control	725/1737	High-resolution melting analysis	0.17	8
Yao et al. [28]	2008	China	Asian	KOA	Case-control	313/485	PCR-RFLP	0.33	8
Vaes et al. [26]	2009	Netherland	Caucasian	KOA	Case-control	667/2097	Taqman	0.72	8
Zhang et al. [5]	2019	China	Asian	KOA	Case-control	288/397	Taqman	0.56	8
García-Alvarado et al. [27]	2018	Mexico	Caucasian	KOA	Case-control	145/145	RT-PCR	0.89	8
Valdes et al.—GOAL [25]	2011	UK	Caucasian	KOA	Case-control	867/758	Allele-specific PCR	0.83	8
Valdes et al.—Hertfordshire [25]	2011	UK	Caucasian	KOA	Case-control	1141/536	Allele-specific PCR	0.22	8
Chapman et al. [11]	2008	Netherland	Caucasian	KOA	Case-control	142/724	Mass spectrometry	0.55	8

### Efficiency analysis

By comprehensive analysis, there is significant relationship between knee osteoarthritis and GDF5 rs143383 polymorphisms. Allele model (C versus T): OR = 0.84 (95% CI = 0.76–0.92); dominate model (CC+CT versus TT): OR = 0.80 (95% CI = 0.72–0.90); recessive model (CC versus CT+TT): OR = 0.79 (95% CI = 0.68–0.92); heterozygote model (CT versus CC+TT): OR = 0.88 (95% CI = 0.80–0.96); homozygous model (CC versus TT): OR = 0.88 (95% CI = 0.80–0.97). Overall analyses are showed in Table 2. OR value of genetic model crosses 1, which means that summary results are not of statistical significance. Summary heterogeneity of genetic model is > 50%, so we conducted subgroup analysis to explain the heterogeneity.

**Table 2:** Meta-analysis for GDF5 rs143383 polymorphisms and knee osteoarthritis risk

Variables	*N*	C vs T		CC+CT vs TT		CC vs CT+TT		CC vs TT		CT vs CC+TT	
		OR (95% CI)	*P*_h_	OR (95% CI)	*P*_h_	OR (95% CI)	*P*_h_	OR (95% CI)	*P*_h_	OR (95% CI)	*P*_h_
Total	20	0.836	1 × 10^−4^	0.80	1 × 10^−4^	0.79	0.001	0.88	0.002	0.88	0.002
		(0.76–0.91)		(0.72–0.90)		(0.68–0.92)		(0.80–0.97)		(0.80–0.96)	
Ethnicity											
Asian	8	0.84	1 × 10^−4^	0.80	1 × 10^−4^	0.76	0.002	0.85	0.011	0.85	0.011
		(0.67–1.02)		(0.63–1.00)		(0.50–1.12)		(0.72–1.00)		(0.72–1.00)	
Caucasian	12	0.84	0.12	0.81	1 × 10^−4^	0.81	0.92	0.90	0.013	0.89	0.016
		(0.79–0.91)		(0.71–0.92)		(0.72–0.90)		(0.80–1.02)		(0.79–1.02)	

*N* number of studies

*P*_h_ value of *Q*-test for heterogeneity test. Random-effects model was used when *p* value for heterogeneity test < 0.05; otherwise, fix-effects model was used

### Subgroup analysis by ethnicity

**Fig. 2:**
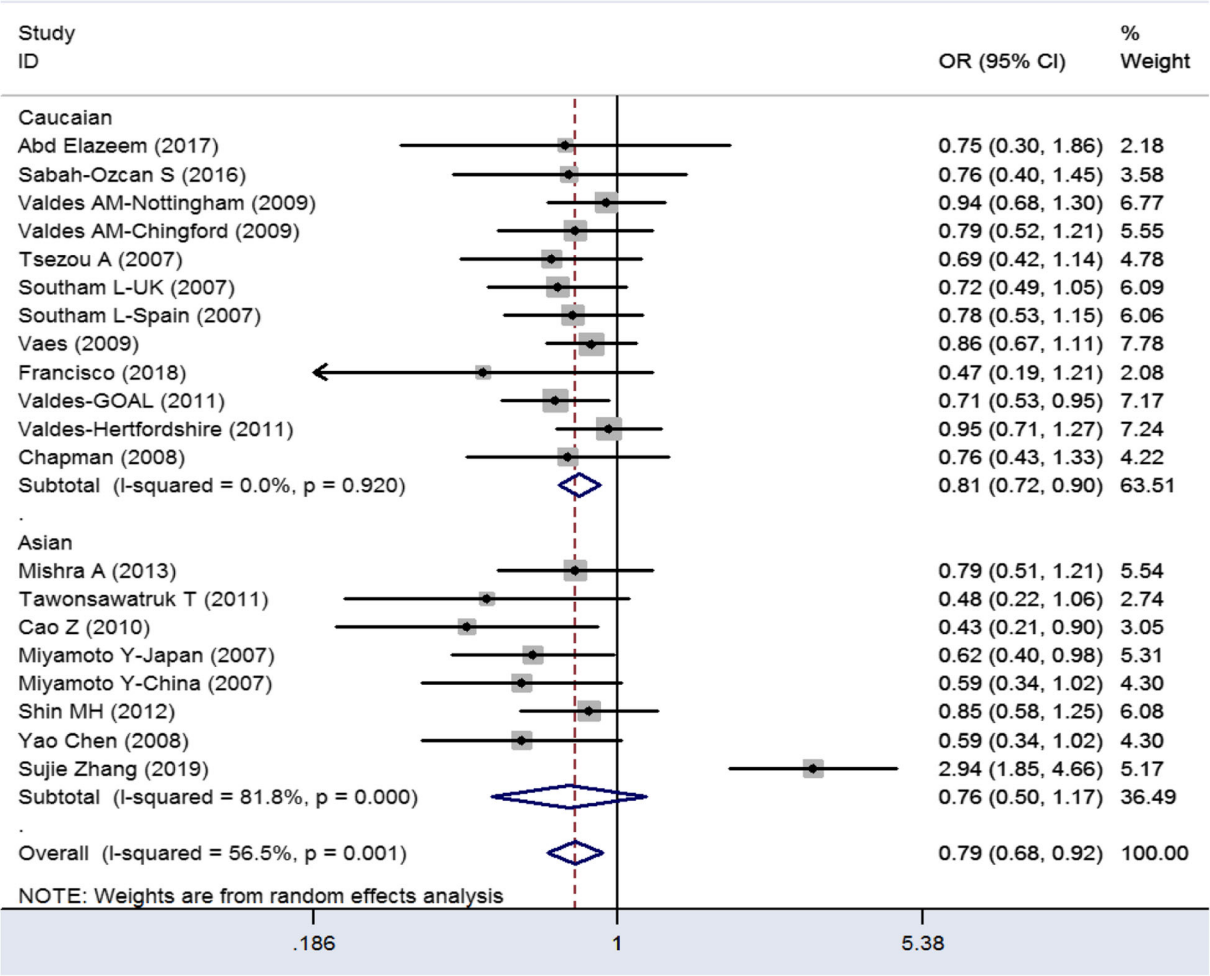
Forest of GDF5 genetic polymorphism (C>T) and KOA (CC vs CT+TT)

**Fig. 3:**
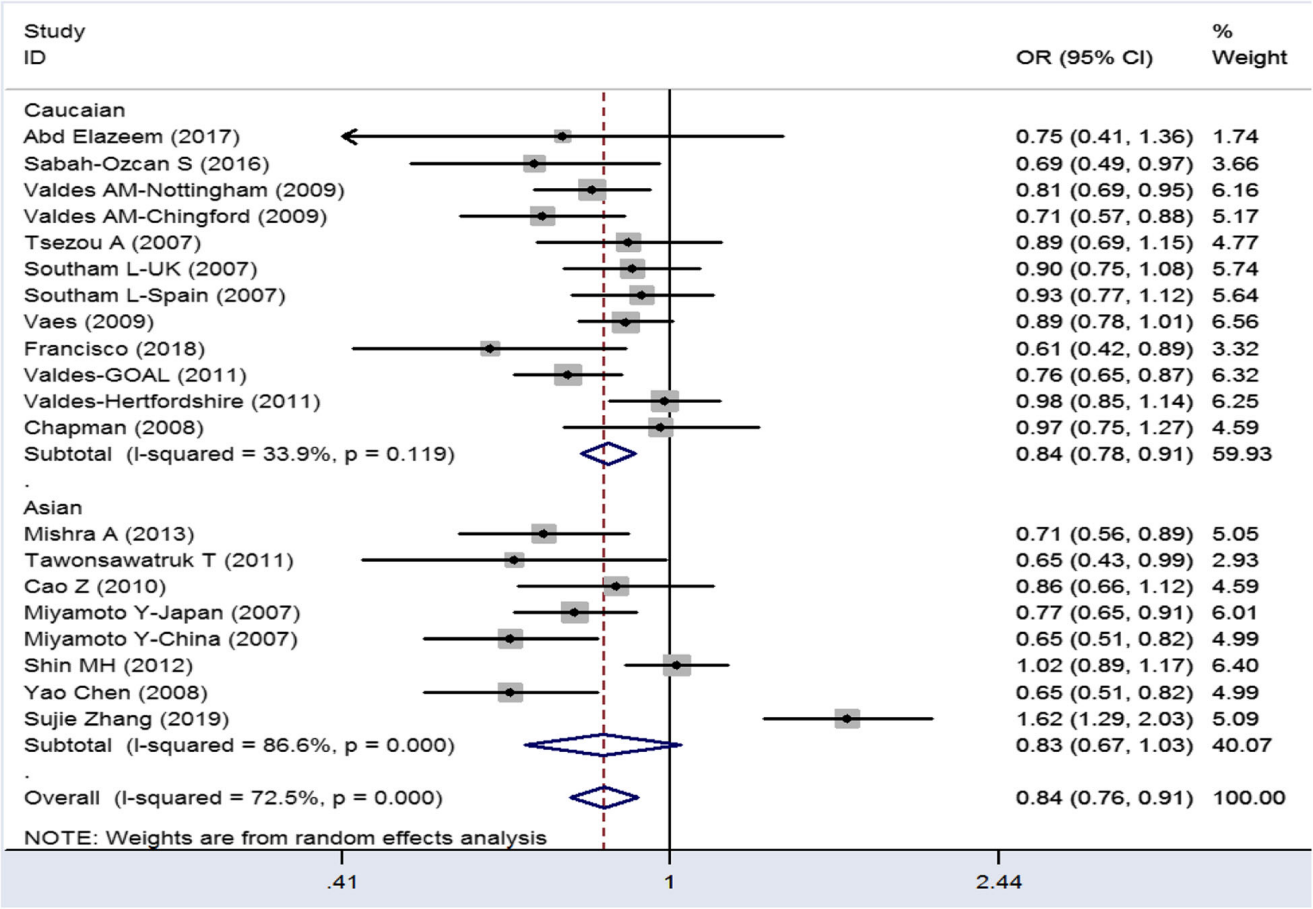
Forest of GDF5 genetic polymorphism (C>T) and KOA (C vs T)

There is significant correlation between knee osteoarthritis and GDF5 rs1433383 polymorphisms in Caucasians. In Caucasians, all genetic model results are showed in Table 2 (C vs. T: OR = 0.79–0.91, *P* < 0.05; CC+CT vs. TT: OR = 0.71–0.92, *P* < 0.05; CC vs. CT+TT: OR = 0.72–0.90, *P* < 0.05; CT vs. CC+TT: OR = 0.79–1.02, *P* < 0.05; CC vs. TT: OR = 0.80–1.02, *P* < 0.05). But in Asians, there is no significant correlation between knee osteoarthritis and GDF5 rs143383 polymorphisms among all genetic model. We did not carry on the subgroup analysis by sex, because previous meta-analysis study conclude sex factor is not significant between GDF5 rs143383 polymorphism and the risk of knee osteoarthritis. A more obvious significant association was exploited for CC vs. TT+CT (OR = 0.81, *P* < 0.05) and C vs. T (OR = 0.84, *P* < 0.05) in comparison with other models in Caucasians (Table 2, Figs. 2 and 3). The results show CC genetic model and C allele are protective factors in KOA. All heterogeneity of genetic model still remains. To solve the heterogeneity, we use random effect model in the meta-analysis and make sensitivity analysis to ensure reliable results.

### Sensitivity analysis and publication Bias

**Fig. 4:**
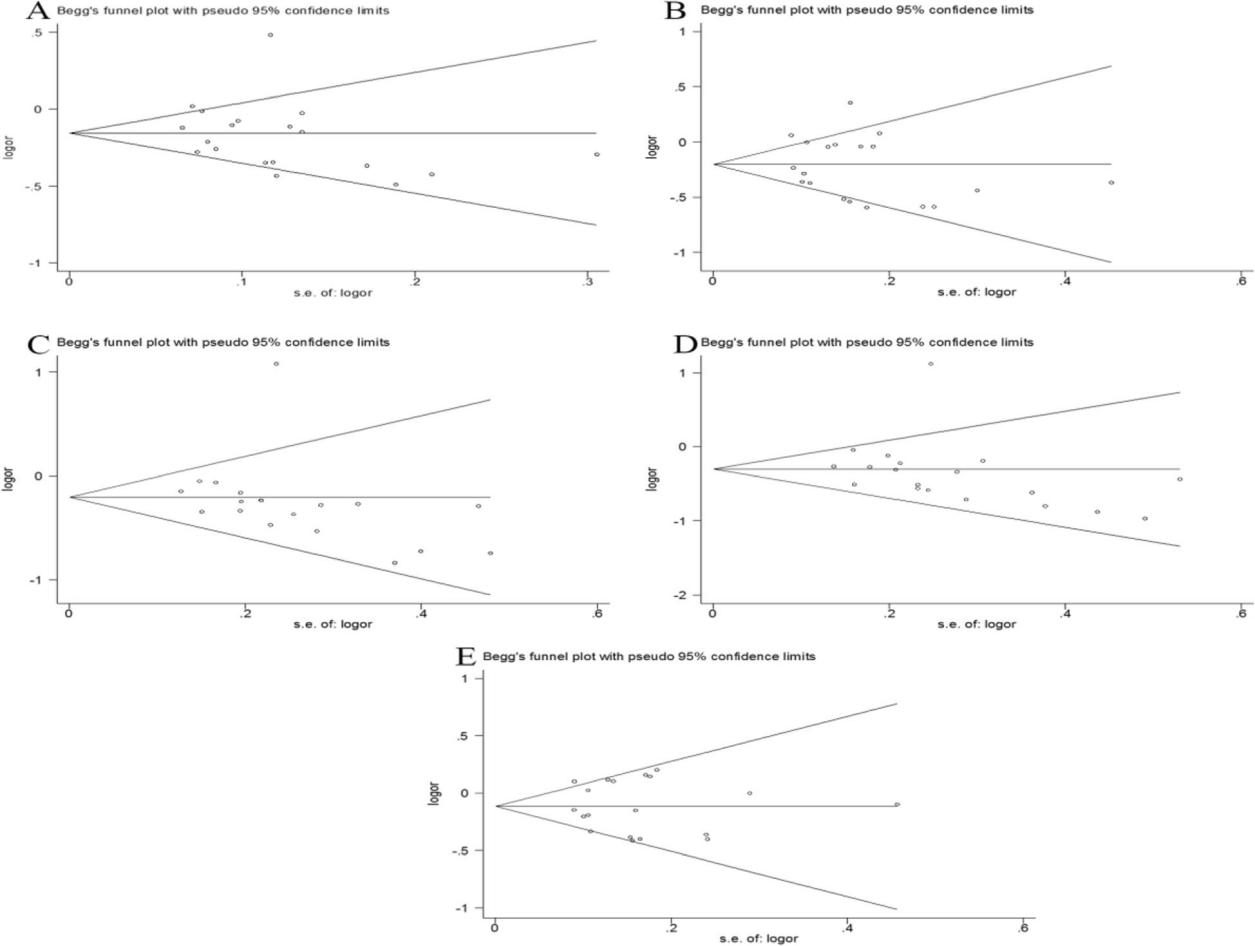
Funnel plots of the association between GDF5 rs143383 polymorphism and knee osteoarthritis (**a** allele model, **b** dominate model, **c** recessive model, **d** homozygote model, **e** heterozygote model)

The stableness of the results is estimated by sensitivity analysis. The sensitivity analysis procedure is a single study deleted from the meta-analysis every time, but the results remain unchanged (Fig. S1). The Begg’s funnel diagram does not show any obvious sign of dissymmetry in all genetic models (Fig. 4). Furthermore, Egger’s regression analysis did not show publication bias in the results (*P* = 0.707 for 2G versus 1G, *P* = 0.452 for 2G/2G versus 1G/1G, *P* = 0.452 for 1G/2G versus 1G/1G, *P* = 0.851 for recessive model, and *P* = 0.133 for dominant model, respectively). Figure 4 and Egger’s regression analysis describe that results are stable and reliable in the current meta-analysis.

## Discussion

In the research article, we firstly found that GDF5 rs143383 polymorphisms affect the risk of knee osteoarthritis in Caucasian but not in Asian, including detailed data from 16 studies in 7997 cases and 12,684 controls. The final results are new observations in previous studies. When carrying on the funnel plot to analyze some bias, the results still are very stable and reliable.

GDF5 is a member of bone morphogenetic proteins (BMP) family, which is located on chromosome 20q11.2 and spans 21.43 kb, from 34,042,573 to 34,021,146 and can form the earliest markers of joint morphology. BMP is an indispensable signal pathway molecule or protein in most kinds of bones [29–37]. There are many cells expressing the GDF5 gene, including articular cartilage, articular capsule, and ligament. GDF5 gene mutation may result in the downregulation of the transcription activity of articular chondrocytes [38–40]. Decreased GDF5 levels in fully formed adult knees may also influence OA risk by impairing homeostasis in healthy joints or by accelerating degeneration due to injury [41]. The growth differentiation factor 5 gene GDF5 was one of the first reported OA susceptibility signals that showed consistent association to OA, with the transcript single nucleotide polymorphism (SNP) rs143383 demonstrating association in Asians and Europeans [42].

On the one hand, previous literatures have said that GDF5 rs143383 polymorphisms C allele is a protective factor for the susceptibility of knee osteoarthritis among Caucasian populations (OR = 0.74, *P* < 0.001) and Asian populations (OR = 0.87, *P* = 0.004) [9]. On the other hand, Zhang et al. holds that interaction of GDF5 rs143383 polymorphisms T allele increases the risk of knee osteoarthritis among Asian (OR = 1.62, *P* < 0.001) [5]. But now our research shows that GDF5 rs143383 polymorphisms are only related to knee osteoarthritis among Caucasian populations by subgroup analysis, not Asian populations.

Although previous meta-analysis literatures thought that high-expressed GDF5 rs143383(C/T) can reduce the risk of knee osteoarthritis, their limitations still remain. Firstly, Pan et al.’s meta-analysis has some shortcomings, including unmatching HWE studies in their meta-analysis [8], which results are unreliable. Secondly, Huang et al.’s meta-analysis explores the association between all kinds of osteoarthritis and GDF5 rs143383 polymorphisms, which cannot precisely describe the relationship between GDF5 rs143383 polymorphisms and the susceptibility of knee osteoarthritis [12]. Although genetic variants can have different effect sizes in different populations, as is shown for GDF5 for African populations vs Eurasian populations [43–45], it is highly unlikely that this is the case, based on the data presented by the authors. These studies focused on apes, so there is still a gap between apes and humans. In the discussion, the authors state that future studies should include more samples and examine more genetic variants. We did not use freely available data from large population association studies (GWAS) which have been published [46–49], because the data of the sample size of the five models cannot be extracted. The meta-analysis mainly focused on GDF5 rs143383, and no other variants of GDF5, because our aim is to solely clarify controversial GDF5 rs143383.

To sum it up, our final results, paralleling to previous results, are more reliable by comprehensive collection and assessment of quality. Furthermore, the association between osteoarthritis and GDF5 is more clear than previous studies.

## Conclusions

Our article has found sufficient pieces of evidences to conclude that the risk of Caucasian’s knee osteoarthritis is the association with GDF5 rs143383 polymorphisms. To our knowledge, this result is the first discovery in which the risk of Asian knee osteoarthritis is not associated with GDF5 rs143383 polymorphisms by meta-analysis. The finding may indicate that GDF5 rs143383 polymorphism mutations are population specific. In the future, the potential study should increase more samples about knee osteoarthritis and more gene variants to precisely and comprehensively clarify the relationship between knee osteoarthritis and gene variants.

## Supplementary information

**Additional file 1: Fig. S1.** Sensitivity analysis of the pooled ORs and 95% CI for the overall analysis.

## Data Availability

The datasets used and/or analyzed during the current study are available from the corresponding author on reasonable request.

## Abbreviations

CI: Confidence interval
HWE: Hardy–Weinberg equilibrium
KOA: Knee osteoarthritis
GDF: Growth differentiation factor
NOS: Newcastle–Ottawa Scale
OA: Osteoarthritis
OR: Odds ratio
BMP: Bone morphogenetic protein
