# Supplementary material for: Association between growth differentiation factor 5 rs143383 genetic polymorphism and the risk of knee osteoarthritis among Caucasian but not Asian: a meta-analysis
Source: Arthritis Res Ther. 2020 Sep 14;22:215. doi: 10.1186/s13075-020-02306-9 (PMC7488690; doi:10.1186/s13075-020-02306-9)
Supplement: Supplementary file 1 — Additional file 1: Fig. S1. Sensitivity analysis of the pooled ORs and 95% CI for the overall analysis. [file 13075_2020_2306_MOESM1_ESM.docx]

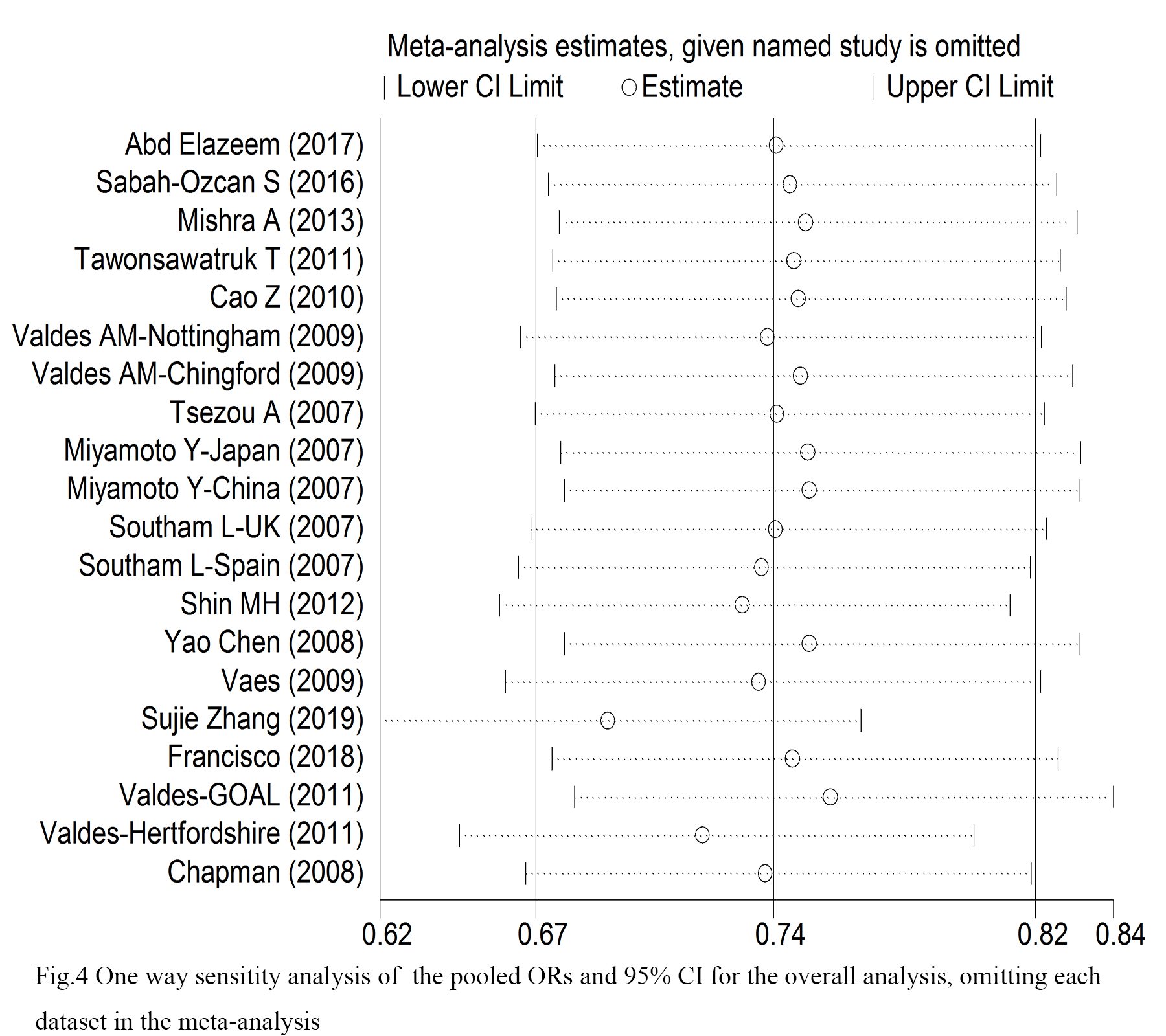


**Fig. S1.** Sensitivity analysis of the pooled ORs and 95% CI for the overall analysis(If the circle value does not exceed the upper and lower limits, the results of this paper are reliable)
